# Delayed Latency of Postural Muscles of Individuals with Intellectual Disabilities

**DOI:** 10.3389/fpsyg.2018.00109

**Published:** 2018-02-06

**Authors:** J. Walter Tolentino-Castro, Andreas Mühlbeier, Luis Mochizuki, Heiko Wagner

**Affiliations:** ^1^Department of Movement Science, University of Münster, Münster, Germany; ^2^Otto Creutzfeldt Center for Cognitive and Behavioral Neuroscience, University of Münster, Münster, Germany; ^3^Department of Movement Science, College of Arts, Sciences and Humanities, University of São Paulo, São Paulo, Brazil

**Keywords:** muscular latency, intellectual disabilities, spinal cord computation, postural control, muscular reflex, spinal cord

## Abstract

Individuals with intellectual disabilities (ID) (50 < IQ < 79) show impaired motor and postural control, these impairments are highly related to falls and injuries. Recent studies demonstrated these impairments are related with fine and gross motor development, which are more strongly associated with cognition, and consequently language for individuals with ID than for without ID. Despite these studies, little is known about the structure and functioning of this population's spinal cord, which is highly involved in postural control. The aim of our study was to assess the latency of the reflex responses in postural muscles after unexpected lateral external perturbations, in individuals with intellectual disabilities compared to typically developed participants. We assessed 16 participants with intellectual disabilities, 9 males and 7 females (aged 24.06 ± 8.66 years) and 20 typical developed participants (CG), 11 females, 9 males, (aged 21.20±1.96 years). While the participants were in an upright standing position electromyography was used to collect data from *M. obliquus externus abdominis* (OE) muscles, which were activated by unpredictable perturbations applied by a servomotor on a hand-held grip, following the lateral external perturbation to the trunk. The intellectual disabilities group presented contralateral OE muscles latency of 85.71±27.24 ms, and CG group presented 68.62±10.25 ms, no differences was found. Ipsilateral OE muscles latency also did not differs between the groups, ID group showed 96.60±30.20 ms and CG group showed 95.57±33.53 ms. Our study furthers the knowledge about the muscular activity of individuals with intellectual disabilities. The present experimental results may suggest unique spinal cord processing of individuals with intellectual disabilities when they are faced with unexpected lateral external perturbations.

## 1. Introduction

Upright posture characterizes most primates, especially hominids. This posture was developed early in human evolution, notably marked by the emergence of *Homo erectus* approximately two million years ago (Niemitz, 2010). Bipedalism, a consequence of this upright posture, is a milestone for human locomotion. While for other primates upright posture is not the most common daily position, for human beings upright posture is largely associated with the activities of daily life. Despite this, postural control is a not easy task, it includes balance in standing, walking and steps transitions but also reaction to perturbations (Wittenberg et al., 2017). Deficits in postural control can lead to dangerous situations, with poor postural control increasing the risk of falls and injuries. This is particularly true for groups of individuals with special needs. In an attempt to reduce the impact or risk of such dangerous situations, a body of research has been dedicated to examining how perturbations affect human postural control.

Individuals with special needs represents a group with high risk of falling and consequent injury (Masud and Morris, 2001; Finlayson et al., 2010). Individuals with intellectual disabilities and poor motor coordination show an elevated risk of falls and poor standing stability (Cherng et al., 2007; Du et al., 2015; Speedtsberg et al., 2017). Individuals with intellectual disabilities have demonstrated deficits in coordination of body movements, difficulties in mastering simple motor activities, including fine and/or gross motor skills, balance and posture (Finlayson et al., 2010; Westendorp et al., 2011; Houwen et al., 2016). Motor performance in such group is usually slower, less accurate, and more variable than that of their peers (Westendorp et al., 2011; Zwicker et al., 2012; Caçola, 2016; Houwen et al., 2016). These deficits can persist into adulthood, and can cause social and emotional difficulties (Mandich and Polatajko, 2003).

The partial absence of nervous system structures, in particular Astrocytes and Oligodendrocytes, which are involved on myelination process, (Kirby et al., 2014; Simons and Nave, 2016) play a role in the motor control of individuals with Down syndrome (Ábrahám et al., 2012). There is evidence that individuals with intellectual disabilities have reduced myelination in the brain, which implicates alterations in structural brain networks (Debrabant et al., 2016). As such, it is possible that, as in Down syndrome, myelination may influence motor control in intellectual disabilities. However, it is not known whether the amount of myelin sheathing in the spinal cord, specific in motor and inter-neurons, of intellectual disabilities individuals is impaired to the extent that it could explain the deficits in the postural control.

It is difficult to measure postural control and spinal cord activation/processing without invasive experimentation. However, we have developed a non-invasive apparatus that produces lateral perturbations, while simultaneously being connected to an Electromyograph that measures the muscular latency of a postural reflex. Recent studies using this apparatus have shown how posture is controlled when uni and bilateral perturbations are applied (Mühlbeier et al., 2017). In addition, chronic low back pain is associated with delayed muscle reflex responses of trunk muscles, which mainly is processed in spinal cord level (Liebetrau et al., 2013). Other study of our group has showed that neural circuits in the spinal cord could substantially contribute to muscular reflex bursting pattern (Wulf et al., 2012). Liebetrau et al. (2013) also suggests that, a delayed muscular reflex latency could have relevant influence on spinal stability, if subjects do not adapt their reflex amplitudes.

Therefore, the aim of the current study was to use the method mentioned above to investigate the latency responses of postural muscles after unexpected upper side perturbation limb loading in typically developed and intellectual disabilities participants. We expected that compared to typical participants, individuals with intellectual disabilities will show delayed (slower) postural control latency after external perturbation. Based on the possibility of poor myelination and atypical processing at the spinal cord level, we also hypothesized that participants with intellectual disabilities will have different latencies and patterns of muscular activity when compared to typically developed individuals, independently of the side of perturbation.

## 2. Methods

### 2.1. Participants

The intellectual disabilities group consisted of 16 participants. All participants under the age of 18 with intellectual disabilities were either currently enrolled schools for people with special needs. All the adults participants of this group were enrolled in employment educational institutions for individuals with intellectual disabilities. The inclusion criteria for intellectual disabilities participants was (50 < IQ < 79), which is the diagnostic criteria for being accept at such schools and institutions, IQ test was made at the acceptance phase of the students/participants with presence of Psychologist expert, class teachers and institution directors. All the control participants were graduate students of Sport Science at University of Münster, any of these participants have historic of any physical injury or intellectual disorder and/or brain abnormalities. The participants and their parents or caregivers received no payment for their participation. For the demographic data of the intellectual disabilities and control sample see Table 1. The study was approved by the research ethics committee of Institute of Psychology and Sport Science of the University of Münster, (2016-12-WTC). Before beginning the experiment the participants and their parents or caregivers were informed about the experimental procedure and written informed consent was given.

**Table 1:** Participants characteristic.

	**ID**	**CG**
Sex (males and females)	(9M/7F)	(9M/11F)
Age (year)	24.06 ± 8.94	21.20 ± 2.01
Height (m)^*^	1.66 ± 0.10	1.74 ± 0.08
Body mass (kg)	70.81 ± 20.90	65.75 ± 10.70
Body mass index ($\frac{m}{kg^{2}}$)^*^	25.21 ± 5.45	21.39 ± 2.14
Applied Force (N)	108.06 ± 32.00	103.20 ± 17.33

* *p < 0.05 means significant difference between the groups*.

### 2.2. Experimental setup and procedure

**Figure 1:**
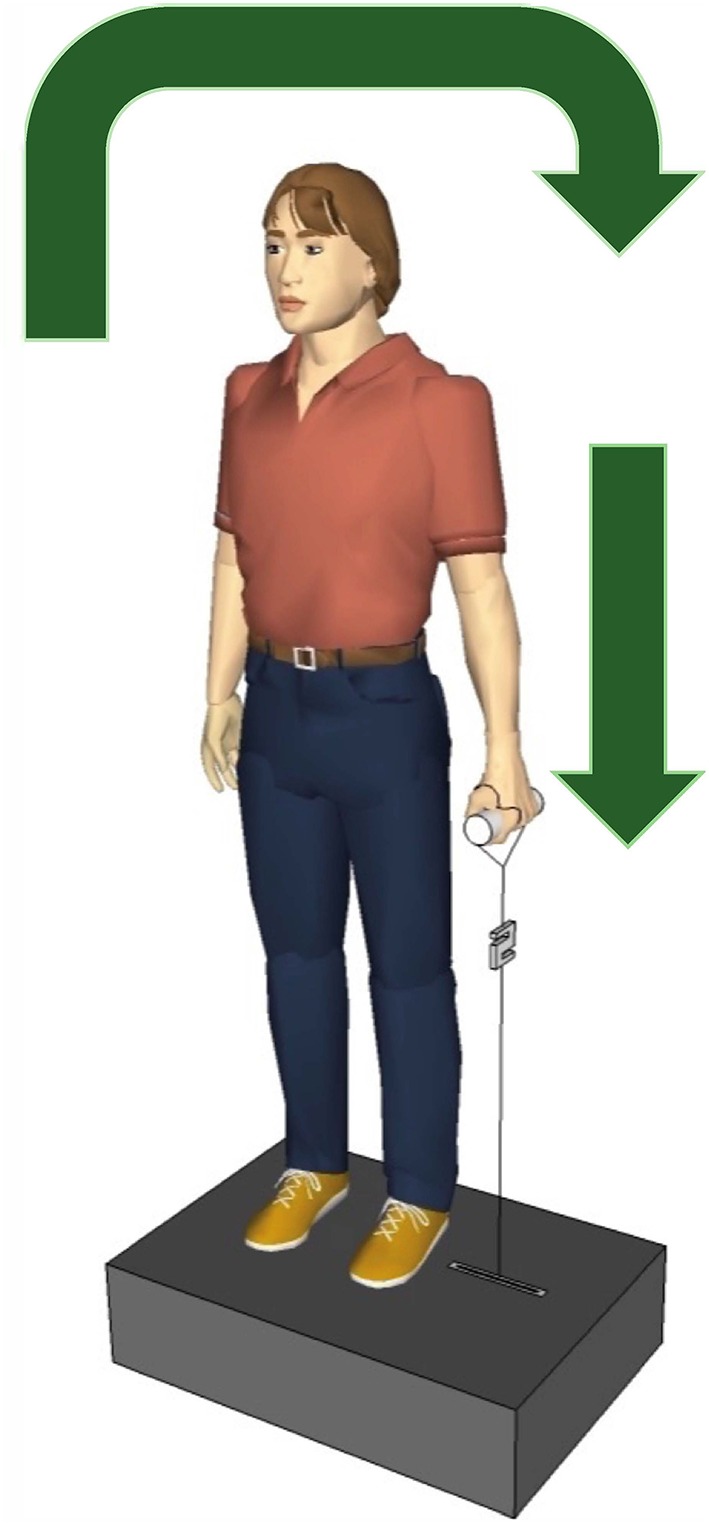
Experimental setup. The participants held the handle in the left and the right hand, one after the other, the servo motor vertically pulled the handle producing a sudden lateral perturbation to the trunk. The arrows show the direction of the body move after perturbation.

The participants were instructed to stay relaxed, looking straight ahead while holding a handle of the apparatus with their right or left hand, with their elbows extended, they knew before the side of the perturbation, but they did know when the perturbations will happens, see Figure 1. The arm, the hand and the lateral malleolus formed an imaginary line. The handle was connected to a servo motor (AKM 44 E, Kollmorgen, Germany; controlled by a Servo Drive S300, Danaher Motion, Germany) via an inelastic string (Dyneema, 1.3 mm, 180 daN, Elliot GmbH, Xanten, Germany). A load cell (50–2000 N, 2 kHz, Biovision, Wehrheim, Germany) was inserted into the string between the handle and the servo motor to measure the force applied to the hand. Twenty lateral perturbations were applied to each participant, ten to the left hand and ten to the right hand, with 3 s interval among the perturbations. The perturbation force was adapted for each participant individually as $F\left[N\right]=16\%\cdot 9.81\frac{m}{s^{2}}\cdot M\left[kg\right]$, based on the participant's body mass M. For example, a participant of *M* = 65 kg was exposed to perturbations with a force of *F* = 102 N. Considering the applied force and duration (100 ms) of the perturbation, the loading was quite abrupt and sudden causing a slight deflection of the participants trunk in the frontal plane. If necessary a correction of the participant's position was given between perturbations.

### 2.3. Electromyography

Surface electromyography signals (sEMG) were recorded (DeMeTec, ToMEMG V1.2, GJB Datentechnik GmbH, Langewiesen, Germany) from the left and right *M. obliquus externus abdominis* (OE) using circular, disposable, double electrodes (H93SG, Ag/Ag-Cl Sensor, Covidien, Neustadt, Germany; diameter 0.5 cm, distance 2.5 cm). The electrode placement followed the SENIAM recommendations (Ng et al., 1997; Hermens et al., 1999). The OE was chosen because in the development of the study it showed the clearest response of the measured trunk muscles (Wulf et al., 2012; Liebetrau et al., 2013; Mühlbeier et al., 2017).

The reference electrode was positioned on the elbow. Due to severe disabilities and the fear of razor blades in several participants from the intellectual disabilities group, a neat preparation of the participants' skin was not always given. For the majority of the participants skin could be shaved and cleaned with medical abrasive paste (OneStep, H+H Medizinprodukte GbR, Münster, Germany). The raw sEMG data were recorded at 2 kHz sampling rate and amplified 2,500 times (ToM Erfassung, GJB Datentechnik GmbH, Langewiesen, Germany).

### 2.4. Data analysis

Force and sEMG time series data was analyzed using MatLab (MathWorks, USA). In every analysis, a probability of less than 0.05 was considered statistically significant (^*^) and less than 0.01 as highly significant (^**^). Since the data for the maximum reflex amplitude and reflex integral revealed high similarity, statistical analysis was limited to the maximum reflex amplitude. So there was no further revision of reflex integral results. Contralateral and ipsilateral muscles were defined according to the perturbation side, e.g., when the pull force was applied to the left handle, the ipsilateral muscles were the left trunk muscles, and the contralateral muscles were the right trunk muscles.

#### 2.4.1. Force

The mechanical side perturbation onset occurred when the force signal reached 10% of the maximum force above the preloading baseline. The muscle latency was the difference between the mechanical side perturbation onset and muscle onset.

#### 2.4.2. sEMG

The sEMG signals were high-pass filtered (4th-order Butterworth filter, 40 Hz), rectified, and smoothed by ± 10 samples moving average. As preview research from our group Liebetrau et al. (2013); Mühlbeier et al. (2017), the reflex onset was defined as the instant at which the signal value exceeded four standard deviations above the average of the preloading baseline activity (400 ms), within the interval of 20–200 ms after response onset. For every participant, the mean value of the 20 ipsilateral (10 left and 10 right) and 20 contralateral (10 left and 10 right) muscle responses was taken into account for the statistical analysis. After that, we generate a mean value of intellectual disabilities and CG groups, we used the mean of all participant of intellectual disabilities and CG groups, for contralateral and ipsilateral sides.

#### 2.4.3. Statistical analysis

A two-way ANOVA were conducted to compare the differences of latencies of the muscles responsible for postural control, *M. obliquus externus abdominis* (OE), for contralateral and ipsilateral sides, after unexpected upper side perturbation limb loading in typically developing and intellectual disabilities participants. The assumptions for the two-way ANOVA were tested by evaluating the fit of the observed data (ipsi and contralateral) to the normal distribution (Kolmogorov-Smirnov test) and the homogeneity of variances (Levene test). To assess difference between groups concerning the muscle latency, we analyzed the means of 20 trials for each side using (IBM SPSS Statistics, Version 22, SPSS Inc, Chicago).

## 3. Results

**Figure 2:**
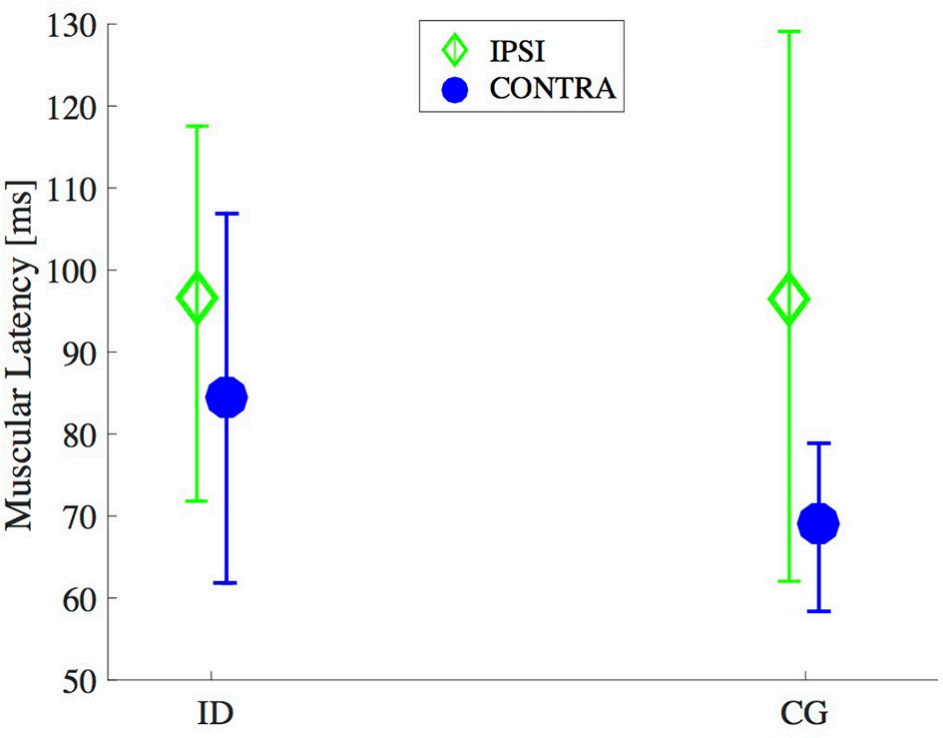
Timing for muscle onset activation after an unexpected external perturbation. Mean and standard deviation of the latency of *M. obliquus* externus abdominis (CONTRA and IPSI sides) of individuals with intellectual disabilities (ID) compared with control group (CG).

Means and standard deviations of the latency of the right and left *M. obliquus* externus abdominis for the groups ID and CG are presented in Figure 2. Two-way ANOVA showed that this muscle latency was affected by the perturbation side (contralateral and ipsilateral) [*F*_(1, 71)_ = 0.8, 0.9, *p* = 0.004] and not affected by group [*F*_(1, 71)_ = 2.0, *p* = 0.15], nor the interaction perturbation side versus group [*F*_(1, 71)_ = 1.6, *p* = 0.20]. The *Post hoc* test showed that the latency of *M. obliquus* externus abdominis was shorter for the contralateral side than for the ipsilateral side (*p* < 0.05).

For ID group, the confidence intervals of 95% was 72.4 and 98.9 ms on contralateral side, for ipsilateral side was 83.3 ms and 109.8 ms. For CG group, the confidence intervals of 95% was between 56.7 ms and 80.5 ms on contralateral side, for ipsilateral side was between 83.6 ms and 107.4 ms.

## 4. Discussion

In this study, we measured the muscular onset after unexpected external lateral perturbations applied to participants with intellectual disabilities and typically developing persons. Our results do not support the hypothesis that the intellectual disabilities group would have delayed muscle activation compared with typically developing persons. Moreover, both groups presented shorter latency for the contralateral side.

The *M. obliquus* externus abdominis is highly related to postural stability. Lateral perturbations induce a specific reaction pattern of the postural regulation, because the contralateral trunk muscle response is faster and has a higher amplitude compared to the ipsilateral muscular response (Wulf et al., 2012; Liebetrau et al., 2013; Mühlbeier et al., 2017); however, participants with intellectual disabilities have not required longer to process changes to the current postural position. This may play a role in critical situations, such as falls or injuries (Gueze et al., 2001; Finlayson et al., 2010). Similar muscle onset suggests that the intellectual impairment may not affect the short latency postural responses. On the other hand, this deviant muscle response pattern has been suggested as factor that predisposes individuals to lower back injuries and may function as a compensatory mechanism to stabilize the spine (Radebold et al., 2000). Muscle recruitment and timing patterns plays an important role in maintaining postural control and lumbar spine stability (Liebetrau et al., 2013). The recovery of postural stability happens fast, usually within a range of 20–200 ms,(Granata et al., 2004) and does not involve the brain. Most computational process required to perceive alterations in one's pose, as well alterations required to regain a stable posture, happens at the spinal cord level via the integration of information from the skin and muscles, through neurons and inter-neurons (Bizzi et al., 1991; Wolpert and Ghahramani, 2000).

As well as being involved in postural control, the spinal cord is heavily involved in other complex motor computations and commands, such as the acquisition and maintenance of motor skills (Bizzi et al., 1991; Kiehn, 2006; Lemon, 2008; Shmuelof and Krakauer, 2012). The spinal cord is a special part of central nervous system, exhibiting functional and structural plasticity, which includes changes in motor neuron firing thresholds, axonal conduction velocity and in synaptic terminals on motor neurons (Wolpaw and Tennissen, 2001; Kiehn, 2006; Lemon, 2008). Consequently, the atypical functioning of the spinal cord is related to an increased risk of falls (Wolpaw, 2007). Our results suggest that intellectual disabilities are not associated with abnormal asymmetrical *M. obliquus* externus abdominis onset (Liebetrau et al., 2013). The mechanisms that might explain why persons with intellectual disabilities have more falls (Cherng et al., 2007; Du et al., 2015; Speedtsberg et al., 2017) is still unknown.

Further research is required to ascertain whether the possible atypical spinal processing in individuals with intellectual disabilities is related to the enhancement of falls and injuries. Although their muscle onset is similar, medular and cortical regulations within the postural control could be different in such populations. If this were the case, it would allow the development of interventions to develop and improve postural control for this population. We suggest that this is particularly important, as people with special needs are known as high risk group of falls, especially as they age (Masud and Morris, 2001; Finlayson et al., 2010). In addition to the research presented here, there are already some indications that individuals with intellectual disabilities have a unique brain signature (Zwicker et al., 2011; Biotteau et al., 2016). This may be related to neuroanatomical abnormalities, specifically a reduction in the amount of myelination and white matter and spine cells function (Ba et al., 2013; Verpelli et al., 2013; Debrabant et al., 2016). We suggest that it is possible that the spinal cord also presents similar abnormalities.

## 5. Limitations of the study

It should be noted that we were able to measured the outcome of the integrative processing between the skin, muscles, motor neurons, inter-neurons and others structures at the surface of the skin, using the EMG Apparatus. As such, we were not able to measure whether whole or part of the spinal cord in individuals with intellectual disabilities is structurally and functionally different from typically developing participants. Even though our study does not provides evidence that such differences may exist; we suggest that more studies are required to confirm or reject this hypothesis.

## 6. Conclusion

The current study furthers the knowledge about how the muscles involved in postural control of individuals with intellectual disabilities works. Our results show no difference of OE muscle latencies in individuals with intellectual disabilities and in typically developed persons. The knowledge about muscle and spinal functioning of persons with ID is useful for fall prevention as well for health care of injuries, and may provide insights for new physical therapies. Interestingly, similar muscle activation are employed for ID individuals compared to CG, independent of the perturbation side. Further research are required to ascertain how posture is controlled in participants with intellectual disabilities.

In summary, we showed that when participants with intellectual disabilities are offered an unexpected lateral external perturbation they take similar time “as typically developing participants” to react to this perturbation. To our knowledge, this is one of the first reports that suggest spinal cord processing of participants with ID is similar to that of typically developing participants. This provides an important starting point for future research into postural control in individuals with ID. Although, there have been prior reports on the atypical postural control of adults (Finlayson et al., 2010; Du et al., 2015) and children with intellectual disabilities (Johnston et al., 2002; Finlayson et al., 2010; Kane and Barden, 2012) we show that unexpected mechanical perturbations did not differ OE muscular latency, compared to CG, which is directly involved in postural control and processed at spinal cord.

## Author contributions

JWT-C and AM made substantial contributions to the conception of the research and interpretation of the data, being also heavily involved in preparing the drafts of the manuscript while HW and LM provided comments on the final revisions. All four authors reviewed and approved of the final manuscript to be submitted. All four authors agree to be accountable for all aspects of the work to ensure that questions related to the accuracy or integrity of any part of the work are appropriately investigated and resolved.

### Conflict of interest statement

The authors declare that the research was conducted in the absence of any commercial or financial relationships that could be construed as a potential conflict of interest.
